# Data-driven predictions and novel hypotheses about zoonotic tick vectors from the genus *Ixodes*

**DOI:** 10.1186/s12898-018-0163-2

**Published:** 2018-02-15

**Authors:** Laura Hyesung Yang, Barbara A. Han

**Affiliations:** 1 Spackenkill High School, 112 Spackenkill Rd., Poughkeepsie, NY 12603 USA; 2 Georgia Institute of Technology, School of Civil and Environmental Engineering, Atlanta, GA 30332 USA; 3 Cary Institute of Ecosystem Studies, Box AB, Millbrook, NY 12545 USA

**Keywords:** Host range, Machine learning, Surveillance, Transmission, Vectorial capacity, Hypostome, Capitulum, *Ixodes*

## Abstract

**Background:**

With the resurgence of tick-borne diseases such as Lyme disease and the emergence of new tick-borne pathogens such as Powassan virus, understanding what distinguishes vectors from non-vectors, and predicting undiscovered tick vectors is a crucial step towards mitigating disease risk in humans. We aimed to identify intrinsic traits that predict which *Ixodes* tick species are confirmed or strongly suspected to be vectors of zoonotic pathogens.

**Methods:**

We focused on the well-studied tick genus *Ixodes* from which many species are known to transmit zoonotic diseases to humans. We apply generalized boosted regression to interrogate over 90 features for over 240 species of *Ixodes* ticks to learn what intrinsic features distinguish zoonotic vectors from non-vector species. In addition to better understanding the biological underpinnings of tick vectorial capacity, the model generates a per species probability of being a zoonotic vector on the basis of intrinsic biological similarity with known *Ixodes* vector species.

**Results:**

Our model predicted vector status with over 91% accuracy, and identified 14 *Ixodes* species with high probabilities (80%) of transmitting infections from animal hosts to humans on the basis of their traits. Distinguishing characteristics of zoonotic tick vectors of *Ixodes* tick species include several anatomical structures that influence host seeking behavior and blood-feeding efficiency from a greater diversity of host species compared to non-vectors.

**Conclusions:**

Overall, these results suggest that zoonotic tick vectors are most likely to be those species where adult females hold a fecundity advantage by producing more eggs per clutch, which develop into larvae that feed on a greater diversity of host species compared to non-vector species. These larvae develop into nymphs whose anatomy are well suited for more efficient and longer feeding times on soft-bodied hosts compared to non-vectors, leading to larger adult females with greater fecundity. In addition to identifying novel, testable hypotheses about intrinsic features driving vectorial capacity across *Ixodes* tick species, our model identifies particular *Ixodes* species with the highest probability of carrying zoonotic diseases, offering specific targets for increased zoonotic investigation and surveillance.

**Electronic supplementary material:**

The online version of this article (10.1186/s12898-018-0163-2) contains supplementary material, which is available to authorized users.

## Background

Ticks transmit a greater diversity of pathogenic agents than any other arthropod [1] and are responsible for vectoring at least 30 zoonotic infectious diseases worldwide [2]. With global warming, tick-borne diseases are projected to increase even more drastically [3]. Unsurprisingly, a large volume of research is dedicated to understanding tick biology with a bias towards a small fraction of tick species known to vector pathogens from animals to humans (zoonotic vectors). Among the hard-bodied ticks (family Ixodidae), the most species-rich genus, *Ixodes*, contains 244 species, of which 29 are known zoonotic vectors (Additional file 1: Table S1). Identifying what intrinsic features distinguish effective zoonotic vectors from non-vector species is essential for understanding the biological drivers of vectorial capacity in *Ixodes* ticks, and for developing preemptive approaches to reducing tick-borne diseases transmitted by *Ixodes* species in humans.

Our goal was to determine which *Ixodes* tick species might harbor undiscovered zoonotic pathogens, and to identify intrinsic biological traits of *Ixodes* ticks that best predict their status as vectors of human zoonotic disease. To achieve this, we applied a machine learning method called generalized boosted regression [4, 5]. This algorithm determines which features are most important for correctly predicting a response variable (here, a binary variable designating whether the *Ixodes* tick species is a zoonotic vector) by building thousands of linked classification trees that successively improve upon the predictions of the previous tree. An advantage of this approach is that it does not rely on distributional assumptions about the data, and is ideal for high-dimensional ecological data containing hidden, nonlinear interactions, and non-random patterns of missing data [6]. In addition to identifying particular *Ixodes* tick species as potentially novel (undocumented) vectors of zoonotic diseases, we describe a suite of features suggesting that zoonotic vectors in this group are species whose anatomy and ecology confer a fecundity advantage that results from more efficient feeding of certain life stages from a broad range of host animals.

## Methods

### Data collection

We recorded a binary score for all 244 ticks of the genus *Ixodes* based on their zoonotic vector status as established by the primary literature and GIDEON database [7], which gives the public health consensus on the status of each *Ixodes* tick species as vector for one or more zoonotic diseases (Additional file 1: Table S1). We first used a standard reference text [19] to standardize Latin binomials for each *Ixodes* species, and to collate data on their biomes and host breadth. For remaining anatomical, biological, and additional biogeographical information across three life stages (larvae, nymph, and adult), we searched peer-reviewed primary literature using the Latin binomial of each species (Additional file 1: Table S2). The references reporting all data used in this study can be found in the Citations column of the data file available in the Figshare repository (10.6084/m9.figshare.3437273). The major goal of this work was to identify traits and testable hypotheses about these traits that enable some species to vector zoonotic pathogens compared to others. Thus, data collection was limited to features describing intrinsic biological differences between species (e.g., anatomy, life history metrics, and biomes); less widely available data related to particular drivers of ecological change (e.g., changing patterns of land use, climate) were excluded.

### Analysis

Using similar approaches applied successfully in previous studies [8, 9], we applied generalized boosted regression via the gbm package in R [4, 5, 10]. We built an ensemble of 30,000 trees using tenfold cross-validation (learning rate = 0.00025, interaction depth = 3; all model parameters reported in Additional file 1: Table S3). Boosted regression methods deal robustly with missing data by the method of surrogate splits, which draws from the correlation structure among trait variables for tree building, effectively treating ‘missingness’ as a feature. In addition, we set a minimum threshold of 1% data coverage across tick species as criteria for inclusion in order to remove variables with near-zero coverage, though the difference between using all traits and removing those with less 1% coverage was negligible to model performance. Data were randomly partitioned into training (70%) and test data (30%). Classification accuracy was measured by area under the receiver operator curve (AUC).

### Study effort

While many epidemiological metrics (e.g., prevalence of tick-borne disease in humans) are biased by study effort (i.e., the amount of healthcare or research spending per country), data on intrinsic features (e.g., tick body size, egg clutch size) are less subject to such biases. However, if study bias across *Ixodes* tick species strongly affects data coverage (i.e., biological features are only known for vectors), study bias can influence model results. To diagnose this possibility, we plotted the probability of a *Ixodes* tick species being a novel vector as a function of citation count, a proxy for study effort (Additional file 2: Figure S1). We also conducted a second boosted regression analysis to determine whether study effort is predictable on the basis of tick species traits (model: citation count ~ intrinsic traits). We found intrinsic traits poorly predicted which *Ixodes* tick species were highly researched (Additional file 1: Table S3). Although *Ixodes* tick species that are known vectors for at least one zoonosis have higher citation counts, there are also several *Ixodes* tick species (vectors and non-vectors) that are reasonably well studied with both low and high probabilities of being zoonotic vectors (Additional file 2: Figure S1). These results confirm that any study bias towards zoonotic vectors does not extend to the tick trait profile reported here, which reflects that of a zoonotic vector rather than that of well studied ticks.

## Results

**Fig. 1:**
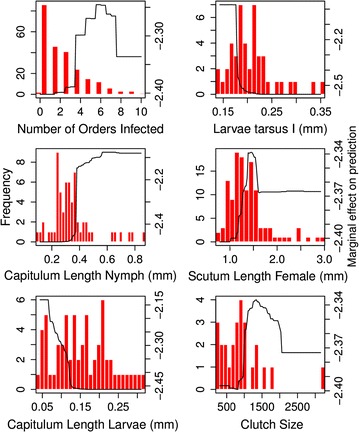
Partial dependence plots representing the top six most important predictor variables from a generalized boosted regression model predicting tick zoonotic vector status. These partial dependence plots illustrate how ticks that transmit zoonotic diseases have distinctive characteristics from non-vectors. In each plot, the frequency histogram represents the trait values for all tick species (vectors and non-vectors) (left y-axis), and the black line shows the marginal effect of each trait on vector status after accounting for the average effects of all other variables in the model (right y-axis). For example, in panel 1, the majority of *Ixodes* ticks parasitize hosts from few taxonomic orders (bars), but tick vectors of zoonotic diseases tend to infest hosts from > 4 orders (line)

Of 244 *Ixodes* species, 29 species are currently recognized as vectors of human diseases in the GIDEON database. Our model was able to distinguish vector from non-vector species with over 91% accuracy on withheld test data. The best predictors of zoonotic vector status included host breadth (number of orders and families that a tick species feeds on); tarsus I length of larvae; capitulum lengths of larvae, nymphs, and female adults; scutum length of female adults; egg clutch size; and female body length (Fig. 1, Additional file 1: Table S3).

Compared to non-vectors, *Ixodes* tick species that vector zoonotic diseases tend to have several distinctive characteristics. First of all, zoonotic vectors have wider host breadth, feeding on host species from five or more families and four or more orders. In addition, the larvae possess shorter tarsus I lengths (the length of the first segment of the first pair of legs; < 0.18 mm), whereas those of non-vectors are generally longer. Larvae and adult female ticks have shorter capitulum lengths than non-vectors, but nymphs exhibit the opposite pattern, with nymphs of zoonotic vector species having longer capitula than non-vectors. Zoonotic vector species were also larger as female adults (scutum length > 1.0 mm, and body length, both while engorged (> 6.0 mm) and unengorged (> 2.5 mm). Adult females of zoonotic vector species also have larger clutches (> 1000 eggs) compared to adult females of non-vector species.

Our model identifies an additional 14 potential *Ixodes* vector species that share similar trait profiles with the 29 species already recognized as zoonotic vectors. Among these, 10 species are reported in primary literature as possibly parasitizing humans (Table 1). The remaining four species, *I. canisuga*, *I. trichosuri*, *I. eldaricus*, and *I. aragaoi*, are novel vectors that have not yet been identified as human parasites but reflect > 80% probability of vectoring a zoonotic pathogen.

**Table 1:** Predicted novel zoonotic *Ixodes* tick vectors ranked in descending order of probability, and citations of primary literature reporting human infestation by each species

Rank	Species	Documentation of human infestation
1	*I. rubicundus*	Horak et al. [25]
2	*I. canisuga*	None
3	*I. acuminatus*	Hillyard [26]
4	*I. vespertilionis*	Piksa et al. [27]
5	*I. sculptus*	Salkeld et al. [28]
6	*I. apronophorus*	Fedorov [29]
7	*I. woodi*	Merten and Durden [30]
8	*I. kingi*	Salkeld et al. [28]
9	*I. kazakstani*	Filippova [31]
10	*I. redikorzevi*	Emchuk [32]; Bursali et al. [33]
11	*I. trichosuri*	None
12	*I. eldaricus*	None
13	*I. laguri*	Bursali et al. [33]
14	*I. aragaoi*	None

A total of 14 tick species were predicted as possible *Ixodes* zoonotic tick species

## Discussion

Identifying *Ixodes* tick species most likely to vector future zoonoses is a critical step toward more effective surveillance and prevention of tickborne disease. Understanding what traits best predict an intrinsic capacity to harbor and transmit zoonotic infections will also facilitate a mechanistic understanding of why some *Ixodes* tick species are better at acquiring and/or transmitting zoonotic infection compared to other species. Here, we report a profile of tick traits that distinguish zoonotic vectors from non-vectors of *Ixodes* tick species with > 90% accuracy. On the basis of these traits, our model identifies particular species with high probabilities of vectoring one or more zoonotic diseases as potential targets for increased investigation and surveillance.

The most important predictor of zoonotic vector status in *Ixodes* ticks was the diversity of vertebrate species parasitized by the tick. This finding is consistent with the general principle that the probability of vectoring a zoonotic disease correlates directly with host range [11, 12]. Several anatomical features were also highly predictive of vector status. Larvae of vector species tend to have shorter tarsus I lengths (length of the first segment of the first pair of legs) compared to non-vectors. The larval stage is important because acquisition of zoonotic pathogens (e.g., Lyme spirochetes) often occurs during the blood meal at this stage [13]. Moreover, if infected at this stage, larvae have two potentially infectious bites through which to transmit pathogens to new hosts, compared to one bite if infected as a nymph. In all three life stages, tarsus I contains many important sensory organs, including Haller’s organ, which promotes habitat-, host-, and mate-seeking behaviors by determining host location, host odors, detecting pheromones, and serving other environmental sensory functions [14]. If the size of Haller’s organ scales allometrically with the length of tarsus I in larvae, tick species with shorter tarsus I lengths than expected for larval body size may indicate a decreased selectivity towards particular host species providing the first blood meal in the tick life cycle. Reduced host selectivity at this stage could lead to more generalized feeding preferences across a wider diversity of host species and environments, increasing the possibility of contact with competent zoonotic hosts that can successfully transmit infection to larval ticks. In a post hoc analysis, we found that ticks with shorter tarsus I lengths at the larval stage indeed fed upon a more diverse host range (infesting hosts from more taxonomic orders) (Additional file 2: Figure S2, *p* = 0.01, *F*_1,43_ = 7.05). This pattern was absent at the other life stages (nymphs: *p* = 0.96, *F*_1,40_ = 0.003; adult females: *p* = 0.82, *F*_1,79_ = 0.05; adult males: *p* = 0.59, *F*_1,53_ = 0.30). In addition to validating comparative patterns across species, future empirical work could explore the relationships between tarsus I and Haller’s organ sizes and their effects on host selectivity in controlled experiments.

Another important trait distinguishing vectors from non-vectors was the capitulum length in larvae, nymphs, and adult females. Capitulum length is determined by hypostome length and salivarium size in ticks. The hypostome is a the ratchet-like anchor within the capitulum that is inserted into the host body [14, 15], and the salivarium is a repository that collects and delivers tick saliva. Tick saliva contains bioactive molecules responsible for facilitating blood meals and can contain zoonotic pathogens such as *Borrelia burgdorferi* (causative agent of Lyme disease) and *Francisella tularensis* (causative agent of tularemia), among others [16]. We found that capitulum lengths were shorter in adult female and larval vectors than those of non-vectors, and that capitulum length in nymphal vectors was longer in zoonotic vectors. This pattern is consistent with widely documented patterns of vector competence of *Ixodes* species that transmit pathogens that cause anaplasmosis, babesiosis, and Lyme disease: of the three developmental stages, the nymphal stage is disproportionately responsible for human transmission [13]. With softer substrates like those encountered in human and other mammal hosts, ticks benefit from a more secure anchor conferred by deeper penetration of mouthparts that comprise the capitulum [15]. Secure attachments lead to increased feeding times, which increase the probability of successful transmission for tick-borne pathogens [15, 16]. Such stage-dependent relationships between capitulum length and feeding behaviors and outcomes (i.e., host breadth, transmission success) present another set of testable hypotheses that, if validated, may suggest that capitulum length at the nymphal stage could be used as an indicator of the vectorial capacity of *Ixodes* tick species for zoonotic pathogens. In contrast, a shorter capitulum in larval and adult tick vectors may signal more generalized feeding that is less selective for particular host species, with host breadth ranging widely across several taxonomic groups.

Our analysis also suggest that *Ixodes* tick vectors may have a fecundity advantage over non-vector ticks [17], with larger adult females producing larger clutches. Specifically, body size, scutum length, and clutch size of adult females were all larger for zoonotic vectors compared to non-vectors. Larger body sizes enable the ingestion of larger blood meals from hosts, leading to greater resources available for egg production [18]. Combined, these results suggest that zoonotic *Ixodes* tick vectors are most likely to be species where adult females produce a larger number of eggs, which develop into larvae that feed on a greater diversity of host species. These larvae may develop into nymphs whose capitula allow for more efficient and longer feeding times on soft-bodied hosts compared to non-vector species, leading to larger adult females with greater fecundity.

Our model identified 14 *Ixodes* tick species that showed ~ 80% probabilities of being undiscovered vectors of zoonotic disease on the basis of their trait similarity with known *Ixodes* vector species (Table 1). The majority of these species reside in Nearctic or Palearctic biomes, and all of them are habituated to forest or grassland habitats [19]. Some of the ticks are suspected in the primary literature as being likely disease vectors, but are not currently recognized by the public health community as zoonotic vectors per se. For example, one species, *Ixodes acuminatus*, is capable of transmitting *Borrelia burgdorferi* sensu lato, though it is not considered an important vector for human disease in nature, perhaps due to infrequent contact with humans [20]. The saliva of another species, *Ixodes rubicundus*, causes paralysis in sheep [21], but to our knowledge there is no record of this species transmitting zoonotic infections to humans. Given that many of the 14 predicted *Ixodes* vectors are understudied and some of them are already suspected to have contact with, and potential health consequences for, humans (Table 1), our study offers new utility for identifying tick species whose intrinsic traits suggest they should be targets for enhanced zoonotic surveillance. In particular, the risk of future tick-borne zoonoses should be monitored in Nearctic and Palearctic regions, which are currently experiencing disproportionately rapid warming [22], and in regions experiencing large-scale ecological changes that are associated with increasing human population densities and declining biodiversity [23, 24]. In addition to informing the biological basis by which some *Ixodes* ticks vector zoonotic pathogens, our study underscores the crucial importance of basic research on ticks and other arthropod vectors, since understanding the biological underpinnings of transmission will rely fundamentally on understanding intrinsic characteristics distinguishing vector from non-vector species of *Ixodes* tick species.

## Conclusions

This study revealed a number of intrinsic biological traits that are highly predictive of zoonotic vector status in the *Ixodes* ticks, and suggest hypotheses about anatomical and biological mechanisms underlying vectorial capacity across *Ixodes* species that now require empirical validation in future work. In general, these distinguishing traits are related to the diversity of hosts species infested by ticks, and suggests that vectorial capacity could be maximized by a suite of features enabling some tick species to feed more efficiently on soft bodied hosts at particular life stages compared to non-vector species. These analyses also reveal several *Ixodes* species that are currently not recognized as vectors of zoonotic disease, but whose biological profile suggests they should be targets of future surveillance.

## Additional files

**Additional file 1: Tables S1–S3.** Tables reporting the zoonotic vector status of all 244 *Ixodes* ticks; A list of all variables include in the boosted regression analysis; and model parameters and performance metrics for boosted regression analyses on vector status and citation count.

**Additional file 2: Figures S1, S2.** Figure S1 shows the relationship between citation count and the probability assigned by a boosted regression model of being a zoonotic vector. Figure S2 shows the relationship between diversity of infested hosts and tarsus I length for *Ixodes* tick species at larval, nymphal, and male and female adult stages.
