# Supplementary material for: Data-driven predictions and novel hypotheses about zoonotic tick vectors from the genus Ixodes
Source: BMC Ecol. 2018 Feb 15;18:7. doi: 10.1186/s12898-018-0163-2 (PMC5815220; doi:10.1186/s12898-018-0163-2)
Supplement: Supplementary file 1 — Additional file 1: Tables S1–S3. Tables reporting the zoonotic vector status of all 244 Ixodes ticks; A list of all variables include in the boosted regression analysis; and model parameters and performance metrics for boosted regression analyses on vector status and citation count. [file 12898_2018_163_MOESM1_ESM.pdf]

Table S1. The zoonotic vector status for all 244 *Ixodes* species. A vector score of 0 indicates that the species does not vector human diseases while a vector score of 1 signifies that the species has been confirmed to transmit human diseases or is strongly suspected to be a vector based on the GIDEON repository (7) and primary literature.

**Species**	**Score**
I. abrocomae	0
I. acuminatus	0
I. acutitarsus	0
I. affinis	1
I. albignaci	0
I. alluaudi	0
I. amarali	0
I. amersoni	0
I. anatis	0
I. andinus	0
I. angustus	1
I. antechini	0
I. apronophorus	0
I. arabukiensis	0
I. aragaoi	0
I. arboricola	1
I. arebiensis	0
I. asanumai	0
I. aulacodi	0
I. auriculaelongae	0
I. auritulus	1
I. australiensis	0
I. baergi	0
I. bakeri	0
I. banksi	0
I. bedfordi	0
I. bequaerti	0
I. berlesei	0
I. bivari	0
I. boliviensis	1
I. brewsterae	0
I. browningi	0
I. brumpti	0
I. brunneus	1
I. calcarhebes	0
I. caledonicus	0
I. canisuga	0

I. capromydis	0
I. catherinei	0
I. cavipalpus	0
I. ceylonensis	0
I. chilensis	0
I. colasbelcouri	0
I. collocaliae	0
I. columnae	0
I. conepati	0
I. confusus	0
I. cookei	1
I. cooleyi	0
I. copei	0
I. cordifer	0
I. cornuae	0
I. cornuatus	1
I. cornutus	0
I. corwini	0
I. crenulatus	0
I. cuernavacensis	0
I. cumulatimpunctatus	0
I. dampfi	0
I. daveyi	0
I. dawesi	0
I. dendrolagi	0
I. dentatus	1
I. dicei	0
I. diomedeae	0
I. diversifossus	0
I. djaronensis	0
I. domerguei	0
I. downsi	0
I. drakensbergensis	0
I. eadsi	0
I. eastoni	0
I. eichhorni	0
I. eldaricus	0
I. elongatus	0
I. eudyptidis	1
I. euplecti	0
I. evansi	0
I. fecialis	0

I. festai	0
I. filippovae	0
I. fossulatus	0
I. frontalis	1
I. fuscipes	0
I. fynbosensis	0
I. galapagoensis	0
I. ghilarovi	0
I. gibbosus	0
I. granulatus	1
I. gregsoni	0
I. guatemalensis	0
I. hearlei	0
I. heinrichi	0
I. hexagonus	1
I. himalayensis	0
I. hirsti	0
I. holocyclus	1
I. hoogstraali	0
I. howelli	0
I. hyatti	0
I. hydromyidis	0
I. jacksoni	0
I. jellisoni	0
I. jonesae	0
I. kaiseri	0
I. kashmiricus	0
I. kazakstani	0
I. kerguelenensis	0
I. kingi	0
I. kohlsi	0
I. kopsteini	0
I. kuntzi	0
I. laguri	0
I. lasallei	0
I. latus	0
I. laysanensis	0
I. lemuris	0
I. lewisi	0
I. lividus	1
I. longiscutatus	0
I. loricatus	1
I. loveridgei	0

I. luciae	0
I. lunatus	0
I. luxuriosus	0
I. macfarlanei	0
I. malayensis	0
I. marmotae	0
I. marxi	1
I. maslovi	0
I. matopi	0
I. mexicanus	0
I. minor	1
I. minutae	0
I. mitchelli	0
I. monospinosus	1
I. montoyanus	0
I. moreli	0
I. moscharius	0
I. moschiferi	0
I. muniensis	0
I. muris	0
I. murreleti	0
I. myospalacis	0
I. myotomys	0
I. myrmecobii	0
I. nairobiensis	0
I. nchisiensis	0
I. nectomys	0
I. neitzi	0
I. nesomys	0
I. neuquenensis	0
I. nicolasi	0
I. nipponensis	1
I. nitens	0
I. nuttalli	0
I. nuttallianus	0
I. occultus	0
I. ochotonae	0
I. okapiae	0
I. oldi	0
I. ornithorhynchi	0
I. ovatus	1
I. pacificus	1
I. paranaensis	0

I. pararicinus	1
I. pavlovskyi	1
I. percavatus	0
I. peromysci	0
I. persulcatus	1
I. petauristae	1
I. philipi	0
I. pilosus	0
I. pomerantzevi	0
I. pomerantzi	0
I. priscicollaris	0
I. procaviae	0
I. prokopjevi	0
I. radfordi	0
I. rageaui	0
I. randrianasoloi	0
I. rasus	0
I. redikorzevi	0
I. rhabdomysae	0
I. ricinus	1
I. rothschildi	0
I. rotundatus	0
I. rubicundus	0
I. rubidus	0
I. rugicollis	0
I. rugosus	0
I. sachalinensis	0
I. scapularis	1
I. schillingsi	0
I. schulzei	0
I. sculptus	0
I. semenovi	0
I. shahi	0
I. siamensis	0
I. sigelos	0
I. signatus	0
I. simplex	0
I. sinaloa	0
I. sinensis	1
I. soricis	0
I. spinae	0
I. spinicoxalis	0
I. spinipalpis	1

I. steini	0
I. stilesi	0
I. stromi	0
I. subterraneus	0
I. succineus	0
I. taglei	0
I. tamaulipas	0
I. tancitarius	0
I. tanuki	0
I. tapirus	0
I. tasmani	0
I. tecpanensis	0
I. texanus	1
I. theilerae	0
I. thomasae	0
I. tiptoni	0
I. tovari	0
I. transvaalensis	0
I. trianguliceps	1
I. trichosuri	0
I. tropicalis	0
I. turdus	1
I. ugandanus	0
I. unicavatus	0
I. uriae	1
I. vanidicus	0
I. venezuelensis	0
I. ventalloi	1
I. vespertilionis	0
I. vestitus	0
I. victoriensis	0
I. walkerae	0
I. werneri	0
I. woodi	0
I. zaglossi	0
I. zairensis	0

Table S2. A complete list of all variables included at the outset of the boosted regression analysis, including definitions and units. Some variables were ultimately excluded from the model on the basis of low coverage or near-zero variation. Included variables can be found in Supplementary Table 3. These data also available in the Figshare repository (DOI: 10.6084/m9.figshare.3437273)

Predictor	Coverage	Definition	Units
Afrotropical	1	(1) Afrotropical: Includes sub-Saharan Africa, Madagascar, islands close to the African continent, and a portion of the Arabian Peninsula (Asia) south to a line running east-west from the Strait of Hormuz to the Red Sea.	NA
Australasian	1	(2) Australasian: Wallaceês Line, which runs between Borneo and Sulawesi and through the Lombok Strait between Bali and Lombok, is considered the north-western limit of this region. All islands east of this line through New Guinea and Australia are included in the Australasian Region, along with New Zealand, the islands between New Guinea and Australia, and the island belt from New Guinea to New Zealand.	NA
Body length		(3) Body length: Length scapular apices to posterior body	mm
a. Male adult	0.38		
b. Unengorged female adult	0.28		
c. Partially engorged female adult	0.22		
d. Fully engorged female adult	0.29		
e. Unengorged nymph	0.2		

f. Partially engorged nymph	0.09		
g. Fully engorged nymph	0.08		
h. Unengorged larvae	0.16		
i. Partially engorged larvae	Data deficient		
j. Fully engorged larvae	Data deficient		
Body width		(4) Body width: The body width of tick species across both sexes and across all life stages when un-, partially or fully engorged.	mm
a. Male adult	0.33		
b. Unengorged female adult	0.23		
c. Partially engorged female adult	0.22		
d. Fully engorged female adult	0.25		
e. Unengorged nymph	0.18		

f. Partially engorged nymph	0.07		
g. Fully engorged nymph	0.07		
h. Unengorged larvae	0.26		
i. Partially engorged larvae	Data deficient		
j. Fully engorged larvae	Data deficient		
Capitulum length		(5) Capitulum length:Length from palpa apices to apices of cornua. Capitulum is the movable anterior extension of the body which includes the palps and mouthparts.	mm
a. Male adult	0.3		
b. Female adult	0.39		
c. Nymph	0.25		
d. Larvae	0.26		
Capitulum width		(6) Capitulum width: Measured at level of cornua	mm
a. Male adult	0.24		
b. Female adult	0.4		

c. Nymph	0.23		
d. Larvae	0.25		
Circumpolar	1	(7) Circumpolar: The poles on polar islands and continental territories	NA
Citation count	1	(8) Citation count: Number of hits when the bionomial of tick was searched on Google Scholar.	NA
Clutch size	0.11	(9) Clutch size: Number of eggs that tick lay.	NA
Dentition tip		(10) Dentition tip: Referring to the arrangement of denticles on the hypostome. Ticks were given numerical value of 1 to 4.	NA
a. Male adult	Data deficient		
b. Female adult	Data deficient		
c. Nymph	0.3		
d. Larvae	Data deficient		
Family	1	(11) Family: Numerical value of number of families that tick infests.	NA
GIDEON	1	(12) GIDEON: Global Infectious Disease and Epidemiology Network. Gave the score of 0 or 1 depending on human infestation description of the bionomial of the tick.	NA
Human infestation	1	(13) Human infestation: A binary indicator describing whether the tick species is known to ectoparasitize humans, according to the Hard Ticks of the World standard reference	NA
Hypostomes length		(14) Hypostomes length: The median ventral extension of the basis capituli, between the palps, and covered with recurved teeth on its ventral surface; with a pronounced preoral canal on its dorsal surface.	mm
a. Male adult	0.24		
b. Female adult	0.35		

c. Nymph	0.2		
d. Larvae	0.23		
Hypostomes width		(15) Hypostomes width: Numerical value of hypostomes width.	mm
a. Male adult	Data deficient		
b. Female adult	Data deficient		
c. Nymph	Data deficient		
d. Larvae	0.14		
Indian ocean	1	(16) Indian ocean	NA
Infects multiple families	1	(17) Infects multiple families: The tick was assigned 1 if it infects more than one family. The tick was assigned 0 if it infects just one family.	NA
Infects multiple orders	1	(18) Infects multiple orders: The tick was assigned 1 if it infects more than one order. The tick was assigned 0 if it infects just one order.	NA
Islands in Central Pacific ocean		(19) Islands in Central Pacific ocean	NA
Islands in the Atlantic ocean		(20) Islands in the Atlantic ocean	NA
Longevity	Data deficient	(21) Longevity: The duration of the life. However, since the experimental conditions varied for many journal articles, it could not be standarized and it was excluded from the analysis.	Days
Metatarsus I length		(22) metatarsus I length: Numerical value of metatarsus I length.	mm
a. Male adult	Data deficient		

b. Female adult	0.2		
Nearctic	1	(23) Nearctic: From the Mexican Plateau northward into Canada and Greenland.	NA
Neotropical	1	(24) Neotropical: From the eastern and western fl anks of the Mexican Plateau southward to southern Argentina and Chile; this region also includes the Greater and Lesser Antilles and the Galapagos Islands.	NA
Number of families	0.98	(25) Number of families: The numerical value of how many other families tick infects. This was extracted from Hard Ticks of the World.	NA
Number of orders	0.98	(26) Number of orders: The numerical value of how many other orders tick infects. This was extracted from Hard Ticks of the World.	NA
Order		(27) Order: The host range of tick. Numerical value of orders that tick infests.	NA
Oriental	1	(28) Oriental: Eastern Pakistan, India, Southeast Asia, southern China and the Ryukyu islands lie within this region, which extends to Wallaceês Line in the southwest (see Australasian Region). The region includes Asia south of the Himalayas in the west and south of the Yangtze River in the east. The western boundary is the Indus River Valley.	NA
Palearctic	1	(29) Palearctic: Europe, Asia north of the Himalayas and west of the Indus River Valley, North Africa through the Sahara, and the northern portion of the Arabian Peninsula (see Afrotropical and Oriental Regions).	NA
Palpi length		(30) Palpi length: Length of the second pair of appendages, normally with 4 segments termed articles.	mm
a. Male adult	0.22		
b. Female adult	Data deficient		
c. Nymph	0.2		
d. Larvae	0.23		
Palpi width		(31) Palpi width: Numerical value of Palpi width.	mm

a. Male adult	Data deficient		
b. Female adult	0.21		
c. Nymph	0.12		
d. Larvae	0.2		
Scutum length		(32) Scutum length: The length of dorsal sclerotized plate covering the anterior part of the body in ixodid ticks	mm
a. Male adult	Data deficient		
b. Female adult	0.5		
c. Nymph	0.27		
d. Larvae	0.26		
Scutum width		(33) Scutum width: Numerical value of scutum width.	mm
a. Male adult	Data deficient		
b. Female adult	0.5		
c. Nymph	0.26		
d. Larvae	0.25		
Spiracular plate diameter	Data deficient	(34) Spiracular plate diameter: The diameter of the spiracular plate, which is a modified plate-like structure on the ventral surface of the body which contains the spiracles. In ixodid ticks, the spiracular plate is a prominent structure with numerous ovoid air spaces within the cuticle which are visible externally.	mm

a. Male adult	Data deficient		mm
b. Female adult	Data deficient		
c. Nymph	Data deficient		
d. Larvae	Data deficient		
Spiracular plate length		(35) Spiracular plate length: The length of the spiracular plate, which is a modified plate-like structure on the ventral surface of the body which contains the spiracles. In ixodid ticks, the spiracular plate is a prominent structure with numerous ovoid air spaces within the cuticle which are visible externally.	mm
a. Male adult	Data deficient		
b. Female adult	0.16		
c. Nymph	0.12		
d. Larvae	Data deficient		
Spiracular plate width		(36) Spiracular plate width: The width of the spiracular plate, which is a modified plate-like structure on the ventral surface of the body which contains the spiracles. In ixodid ticks, the spiracular plate is a prominent structure with numerous ovoid air spaces within the cuticle which are visible externally.	mm
a. Male adult	Data deficient		
b. Female adult	0.14		
c. Nymph	0.11		
d. Larvae	Data deficient		

Tarsus I length		(37) Tarsus I length: The length of the terminal segment of leg I, which contains Haller's organ on dorsal surface as well as other complex sensory structures.	mm
a. Male adult	0.23		
b. Female adult	0.34		
c. Nymph	0.17		
d. Larvae	0.18		
Tarsus I width	Data deficient	(38) Tarsus I width: The width of the terminal segment of leg I, which contains Haller's organ on dorsal surface as well as other complex sensory structures.	mm
a. Male adult	Data deficient		
b. Female adult	Data deficient		
c. Nymph	Data deficient		
d. Larvae	Data deficient		
Tarsus IV length		(39) Tarsus IV length: The length of the terminal segment of leg IV	mm
a. Male adult	Data deficient		
b. Female adult	0.34		
c. Nymph	0.15		
d. Larvae	Data deficient		
Tarsus IV width	Data deficient	(40) Tarsus IV width: The width of the terminal segment of leg IV	mm

a. Male adult	Data deficient		
b. Female adult	Data deficient		
c. Nymph	Data deficient		
d. Larvae	Data deficient		
Zooregion diversity	1	(41) Zooregion diversity: If the tick inhabited more than one of zoogeographical region, it was given the score of 1. If the tick inhabits only in one region, it was assigned 0.	NA

Table S3. The parameters, performance, and results of two generalized boosted regression models: the first model (Vector status) describes the traits predicting zoonotic vector status (the response variable) of tick species from the genus *Ixodes*. The second model was constructed to investigate how well traits of *Ixodes* ticks predict the study effort dedicated to each tick species (CitCount, the response variable; CitCount = the number of citations returned from a search on the Latin binomial for each of 244 *Ixodes* tick species in Web of Science). These results show high accuracy (0.916) for the model predicting vector status. In contrast, study effort is poorly predicted by tick traits (low pseudo-R^2 on test data), suggesting that any sampling bias that exists for *Ixodes* tick species (Supplementary Figure 1) does not extend to tick traits.

**Response**	**Vector status**		**CitCount**	
**Error distribution**	bernoulli		poisson	
**Train/test fraction**	70/30		70/30	
**Shrinkage**	0.00025		0.00015	
**Interaction depth**	3		3	
**Best.iter**	9645/30,000		29745/50,000	
**Performance (train)**	0.988 (AUC)		0.613 (pseudo-R2)	
**Performance (test)**	0.916 (AUC)		-0.058 (pseudo-R2)	
	**Predictive Traits**	**Rel. Imp.**	**Predictive Traits**	**Rel. Imp.**
	Capitulum length larvae	10.563	Clutch.size	65.790
	Number of families infected	10.445	Body width unfed nymph	7.323
	Number of orders infected	10.184	Body width unengorged adult female	5.574
	Multiple orders infected	9.178	Capitulum length larvae	5.507
	Zooregion Diversity	8.911	Body length fully engorged nymph	4.894
	Clutch.size	3.895	Body length unengorged adult female	4.127
	Body length adult male	3.148	Body length unfed nymph	2.630

Body length fully engorged female	3.131	Palpi width larvae	1.584
Capitulum length adult female	2.702	Body length fully engorged female	1.263
Scutum length adult female	2.685	Body width unfed larvae	0.546
Body length unengorged adult female	2.542	Bod width fully engorged female	0.313
Palpi length larvae	2.256	Capitulum width larvae	0.156
Body width adult male	2.130	Tarsus I length larvae	0.074
Tarsus I length larvae	2.090	Number of orders infected	0.033
Palearctic	2.043	Number of families infected	0.031
Body length unfed nymph	1.713	Body length unfed larvae	0.023
Tarsus IV length female	1.576	Multiple orders infected	0.022
Scutum width adult female	1.568	Scutum width larvae	0.020
Oriental	1.527	Multiple families infected	0.018
Afrotropical	1.524	Palpi length larvae	0.009
Capitulum length nymph	1.351	Zooregion Diversity	0.007
Palpi width larvae	1.278	Body width adult male	0.005
Body length unfed larvae	1.157	Hypostome length larvae	0.005
Capitulum width larvae	1.073	Scutum length adult female	0.004
Nearctic	1.038	Hypostome length nymph	0.003
Multiple families infected	0.940	Capitulum length nymph	0.003
Bod width fully engorged female	0.935	Scutum length nymph	0.003
Scutum length larvae	0.803	Scutum length larvae	0.003

Palpi length nymph	0.772	Capitulum width nymph	0.003
Tarsus I length adult male	0.638	Palpi length nymph	0.003
Capitulum length adult male	0.528	Palearctic	0.003
Hypostome length larvae	0.504	Body width fully engorged nymph	0.002
Tarsus I length female	0.498	Capitulum length adult female	0.002
Scutum width larvae	0.489	Oriental	0.002
Body width unfed larvae	0.488	Scutum width adult female	0.002
Body length fully engorged nymph	0.448	Capitulum width adult female	0.002
Capitulum width adult male	0.326	Body length adult male	0.002
Neotropical	0.326	Hypostome length adult female	0.001
Body length partially engorged nymph	0.319	Hypostome length adult male	0.001
Spiracular plate width nymph	0.317	Scutum width nymph	0.001
Spiracular plate length nymph	0.307	denition tip nymph	0.001
Spiracular plate length female	0.227	Tarsus I length adult male	0.001
Scutum width nymph	0.138	Palpi length adult male	0.001
Body width unfed nymph	0.130	Tarsus IV length female	0.001
Body length partially engorged female	0.123	Body length partially engorged nymph	0.001
Body width unengorged adult female	0.106	Tarsus I length nymph	0.001
Tarsus I length nymph	0.102	Capitulum width adult male	0.001
Body width partially engorged female	0.094	Nearctic	0.001
denition tip nymph	0.082	Tarsus I length female	0.000

Capitulum width adult male	0.076	Capitulum length adult male	0.000
Palpi length adult male	0.076	Afrotropical	0.000
Hypostome length adult female	0.071	Spiracular plate length nymph	0.000
Capitulum width nymph	0.070	Tarsus IV length nymph	0.000
Hypostome length adult male	0.068	Spiracular plate width nymph	0.000
Spiracular plate length nymph	0.057	Australasian	0.000
Tarsus IV length nymph	0.055	Hypostome width larvae	0.000
Australasian	0.040	Body width partially engorged nymph	0.000
Spiracular plate length female	0.029	Neotropical	0.000
Body width partially engorged nymph	0.021	Spiracular plate length female	0.000
Hypostome width larvae	0.020	Spiracular plate width female	0.000
Hypostome length nymph	0.019	Palpi width nymph	0.000
Body width partially engorged nymph	0.018	Body width partially engorged female	0.000
Palpi width female	0.013	Metatarsus I length adult female	0.000
Palpi width nymph	0.012	Body length partially engorged female	0.000
Metatarsus I length adult female	0.004	Palpi width female	0.000
Denition base nymph	0.000	Denition base nymph	0.000

Table S4. Full citations for references cited in Table 1 reporting human infestation by predicted zoonotic tick vectors.

Rank	Species	Documentation of Human Infestation
1	*I. rubicundus*	Horak, I. G., Fourie, L. J., Heyne, H., Walker, J. B. & Needham, G. R. Ixodid Ticks Feeding on Humans in South Africa: with Notes on Preferred Hosts, Geographic Distribution, Seasonal Occurrence and Transmission of Pathogens. *Exp. Appl. Acarol.* **27,** 113–136 (2002).
2	*I. canisuga*	None
3	*I. acuminatus*	Hillyard, P. Ticks of north-west Europe—keys and notes for identification of species. Synopses of the British fauna no. 52. *Field Studies Council, Shrewsbury, United Kingdom* (1996).
4	*I. vespertilionis*	Piksa, K., Nowak-Chmura, M. & Siuda, K. First case of human infestation by the tick Ixodes vespertilionis (Acari: Ixodidae). *Int. J. Acarology* **39,** 1–2 (2013).
5	*I. sculptus*	Salkeld, D. J., Eisen, R. J., Antolin, M. F., Stapp, P. & Eisen, L. Host usage and seasonal activity patterns of Ixodes kingi and I. sculptus (Acari: Ixodidae) nymphs in a Colorado prairie landscape, with a summary of published North American host records for all life stages. *J. Vector Ecol.* **31,** 168–180 (2006).
6	*I. apronophorus*	Fedorov, V. G. [Ixodoidea ticks on men in Western Siberia]. *Med. Parazitol.* **37,** 615–616 (1968).
7	*I. woodi*	Merten, H. A. & Durden, L. A. A state-by-state survey of ticks recorded from humans in the United States. *J. Vector Ecol.* **25,** 102–113 (2000).

8	*I. kingi*	Salkeld, D. J., Eisen, R. J., Antolin, M. F., Stapp, P. & Eisen, L. Host usage and seasonal activity patterns of Ixodes kingi and I. sculptus (Acari: Ixodidae) nymphs in a Colorado prairie landscape, with a summary of published North American host records for all life stages. *J. Vector Ecol.* **31,** 168–180 (2006).
9	*I. kazakstani*	Filippova, N. A. *Ixodid ticks (Ixodinae). Fauna USSR New Ser. 4 (4)*. (Nauka, Moscow, Leningrad, 1977).
10	*I. redikorzevi*	Emchuk, E. M. *CERTAIN BIOLOGICAL PECULIARITIES OF IXODES REDIKORZEVI OLEN*. (1968).<br><br>Bursali, A., Keskin, A. & Tekin, S. A review of the ticks (Acari: Ixodida) of Turkey: species diversity, hosts and geographical distribution. *Exp. Appl. Acarol.* **57,** 91–104 (2012).
11	*I. trichosuri*	None
12	*I. eldaricus*	None
13	*I. laguri*	Bursali, A., Keskin, A. & Tekin, S. A review of the ticks (Acari: Ixodida) of Turkey: species diversity, hosts and geographical distribution. *Exp. Appl. Acarol.* **57,** 91–104 (2012).
14	*I. aragaoi*	None
