# Supplementary material for: Data-driven predictions and novel hypotheses about zoonotic tick vectors from the genus Ixodes
Source: BMC Ecol. 2018 Feb 15;18:7. doi: 10.1186/s12898-018-0163-2 (PMC5815220; doi:10.1186/s12898-018-0163-2)
Supplement: Supplementary file 2 — Additional file 2: Figures S1, S2. Figure S1 shows the relationship between citation count and the probability assigned by a boosted regression model of being a zoonotic vector. Figure S2 shows the relationship between diversity of infested hosts and tarsus I length for Ixodes tick species at larval, nymphal, and male and female adult stages. [file 12898_2018_163_MOESM2_ESM.docx]

**Additional Figures**

**Figure S1.** Scatterplot of the total number of citations recorded from a search conducted in Web of Science on the Latin binomial for each *Ixodes* tick species on a log scale (log(citation count)) vs. the probability of being a zoonotic vector (black circles) assigned to each tick species by a boosted regression model. Species that are currently recognized as zoonotic vectors are red triangles. This plot shows evidence that tick species that are recognized to be zoonotic vectors have many citations records. It also shows that there are some tick species that are not well studied despite being zoonotic vectors. There are also several species predicted to be novel zoonotic vectors that are better studied than confirmed vector species. This plot shows that while study effort is, unsurprisingly, greater for zoonotic vector species, it is not a categorical bias influencing the analysis predicting novel vector species.

**Figure S2.** Relationships between host range (here, approximated by the number of taxonomic orders infested by each tick species) and the length of the first segment of the first pair of legs (tarsus I) in larval, nymphal, and female and male adult ticks. These plots show no relationship, except for a statistically significant negative relationship where larvae with small tarsus I lengths infest a greater diversity of host species.
